# The Medical Profession

**Published:** 1847-04

**Authors:** 


ART. V.—*The Medical Profession.*

As the facilities for conveying uttered thought are multiplying, it is truly a source of gratulation to those who have the ennobling interests of our profession at heart, to have thus brought near the most remote cultivators of our art, and honest endeavor strengthened and encouraged by repetition of assurances of a common professional unity and sympathy. Present events have contributed to this in great degree. For not only have we had brought to our own door "Lectures introductory" to the assembled students of the recently opened Medical University in this City, inculcating the necessity for a high standard of medical education—breathing the hope, and promising by earnest endeavor so to raise it, while at the same time they set forth its attractions and fitting incentives for laborious application—but there have come also out of the East and from the far West,as by echo,the re-affirming of like sentiments. Thus at the East and West, North and South, from every quarter, the strong in mind and heart are found upraising, first themselves, and then, by their genial spirit, quickening the minds and hearts of others. Never was there more need, for never was there a time when so many in the profession, and the young especially, were so depressed with forebodings by sight of the accumulated and accumulating wind-clouds which threaten to inundate, undermine, and erase the land-marks of our profession. With repetition of occasions which call for utterance from those who have gained position in community, and whose voices may thus be heard loud above the tumultuous uproar of charlatanry—as on the seacoast by providential foresight is stationed the heavy artillery whose "boom" lifts the mists from the troubled waters, and gives timely warning of the strand—thus all are led to thank God, and take courage.

These and like reflections were called forth more especially by reading the following graphic description of our profession, and its votaries, as found in a secular paper, the St. Louis Union, being a sketch of the remarks made by Dr. Linton, and called forth by a toast drank at the recent celebration of the 83d anniversary of the settlement of that City : .

In arising on an occasion like this, to respond in behalf of the medical profession, I acknowledge that I feel a weight of responsibility, which at the same time inspires me with a degree of pride and a still greater degree of humility—pride in its mighty name and achievements, humility in my own inadequacy to speak of them as they deserve. If the knowledge of the organization and functions of creation's master-piece—man—together with the laws which war against its well-being, and the agencies calculated to promote it, deserves the name of learning and philosophy, the medical profession is a learned profession, and medicine a most important branch of philosophy.

A slight acquaintance with the nature of medical researches, is sufficient to convince any one that they are profound and difficult.

In arriving at conclusions in mathematics, only a few unvarying facts and circumstances have to be estimated. The same; in a great degree, I take to be the case with the law. The same of the mechanic arts, the circumstances are fixed, the facts unvarying from which conclusions are deduced. Simple facts constituted the keys with which Newton unlocked the startling and stupendous mysteries of the starry heavens and was enabled to weigh, as in balances, the ponderous world, spoken into being and heaved into harmonious and eternal motion by the Omnipotence of God—a few simple and unvarying circumstances enable the astronomer to mark the aphelion and perihelion and eclipses of the planets—tell how long comets will be gone on their stupendous journeys, and when they will blaze again in the sight of earth born mortals, "perplexing monarchs,"and awakening fear, superstition and wonder. A simple calculation enables us to tell the distance from the crust of the earth to the boiling lava of its interior—*not so medicine*. It is a deeper, a more difficult, a sublimer and more beneficient study than these. It is deeper—it looks with its microscope into the dizzy abyss of the blood globule, and the elementary fibre—marks the unerring mutations of atoms invisible to the naked eye—and sees phenomena as astounding as those which meet the upturned eye of the astronomer as trains of stars and planets pass in silent and solemn pomp before the wide sweep of his telescope. With his delicate chemical tests he fixes the character of atoms that constitute the "lower deeps" of human organization and nature, and like a second ADAM, gives names to those various classes of invisible constituents of that world within itself—that microcosm, the human system. He dissects every fibre, every nerve, every vessel—he pores over the deep riddle of every function—he looks abroad upon the thousand sources whence flow the well being of man—the thousand causes which constantly tend to mar the adoptation of his organism, and the harmony of his movements, and the thousands of agents which the power of art can oppose to these deleterious causes. But this being is ever changing; these agencies which are on him, are ever fluctuating—man is not to-day what he was yesterday or will be to-morrow. The medicine that acted kindly an hour ago, may be insufficient now and injurious an hour hence—all is fluctuation and change. These changes it is true, take place in accordance with laws as fixed as the pillars of Heaven, but the facts, the *circumstances* by which they are to be estimated, are so numerous and often so obscure, that the stoutest intellect and the most unflagging attention are scarcely able to follow them through their everlasting metamorphoses. It is like calculating and predicting the movements, the shapes, and the colors of the fleecy clouds which float in the heavens—and yet the strong-minded and observant physician calculates these changes of man and modifies them with a precision apparently superhuman.

This is a sublime study. It embraces not only the wonders of man as a material being, but also the mind of man—that which with the quickness of the lightning's flash traverses the sad yet pleasing empire of memory, or the far off enchanting illusory region of

Hope—that which thinks, reasons, loves—that high born emanation from God, compared with which the astonishing magnificence of unintelligent creation is poor indeed.

The medical, is a philanthropic profession. It responds as an angel of mercy to the cries and groans of a suffering world. Who visits the hovel to calm the pains of the beggars? The doctor. Who attend the thousand hospitals of the civilized world, and often without compensation? Physicians. Who bends over and soothes the outcast over whom Death shakes his dart? The medical man. Who follows the army in its march and is seen on the field of battle endeavoring to repair the havoc of war! The Doctor. Sir, the Doctors are a considerable sort of men! They have done the world some service, and they are destined to do a great deal more. They have not yet exhausted the vast study I have attempted to delineate. They are on a grand voyage of discovery in quest of richer treasures than the golden fleece of the Argonauts. They are in sight of some of the distant mountain-tops of the El Dorado; and humanity will yet rejoice with exceeding joy, that Heaven ever gave Physicians to the world, as some mitigation of the curses of original sin. They have already done a great deal, and I could speak of a Harvey, who discovered the circulation of the blood—a Jenner, who by the introduction of vaccination, disarmed of its terrors one of the direst scourges of the world—a Stromeyer and Diffenbach, who discovered the modes of rectifying deformities, before their time regarded as irremediable—of a Civiale, who has substituted a mild for a horrible mode of ridding the system of one of its most deplorable conditions. I might go on with the list, but time does not permit.

I know that it is no strained eulogy to say that Physicians have always been amongst the most intelligent, learned and philanthropic of their country and their age—they have furnished every country a charm at once.

Look at the past—at old Hipocrates, Galen, Celsus, whose writings are yet cherished by us, and whose memories are fresh and blooming, though twenty centuries have swept by with all their revolutions since they lived. Look at a Larrey, a Dupuytren, Cooper, Rush, Physic, who have within the last few years, laid down to sleep the sleep of death, after life's fitful fever. Centuries hence they will be revered for their learning and their labors—look at the living galaxy, Andral, Velpeau, Brodie, Liston, Williams, Elliotson, Mott, Dunglison, Horner, and hundreds of others; their names will never perish. They will rest from their labors—but their good works will forever follow humanity to mitigate its woes.

But I should say something of the physicians of our own glorious city. St. Louis has no reason, I humbly conceive, to be ashamed of her faculty. The quantity, at any rate, cannot be complained of. She has enough Doctors, I suppose, and as far as my acquaintances extend, they are intelligent, studious, conscientious—I don't include the quacks, reccollect? I am proud to call them brethren (the quacks excepted, mind!) and doubt not that the names of many of them will live in the pages of history. They are an eminently decent set of men; if there be exceptions, they are not numerous enough to destroy the rule.

The rapid increase of wealth and population which has blessed St. Louis, has given an impulse to every interest and movement.—The physicians have contributed their full portion of this influence—they study as hard and to as much purpose as physicians anywhere else. Our book stores furnish them with the new works as fast as they are published, so that they are not behind the times. They sustain two Medical Journals. Take them any way, and you will find them *au fait*.

Such is the work—such are the men, who are banded together, not in St. Louis only, not in our country only, but throughout the world—for some there are doing missionary work in heathen lands in behalf of their profession and suffering humanity—laboring with a deep conviction that in the hands of the most cultivated and most gifted, the study and successful practice of medicine requires all their devotedness. That much is yet hidden from human ken, they feel conscious, but this does not dampen, but serves to stimulate their ardor. They believe not in any royal, short, or "patented" road that shall make "doctoring" plain and easy in a few short and simple lessons, or that the human organism is a riddle whose mysteries are to be revealed by shrewdness of Yankee "guesses," but on the contrary, the law of our being, here, as elsewhere holds good; and work and toil, toil and work alone are adequate to penetrate and fathom its depths profound. And, while the characteristics of disease are mutable, yet obeying their own law, no one article of medicine with all its vaunted potency, or its many articles in the hands of the unskilled, can be made to subserve these changes of condition.

The address as quoted above is full of suggestive thought which crowds upon the mind of the reader, and with such sentiments entertained and inculcated we are not one of those who despond in view of the prospective future, how numerous soever Medical Colleges may become, and though the aggregate of students, per Catalogue, are numbered by thousands, and though occasionally an Abernethy, in view of his own large class, shall be caught thinking aloud, "God bless you, I do not know what will become of all of you," of this we feel assured: their accumulated intelligence will not be lost to the community, if by the increased facilities for education we have multiplication of learned physicians, though their multiplication may not enable the keenest sighted to see what will become of *them*, the general intelligence of the community necessarily they will be elevated whether *they* are thrown back upon it or remain in the ranks of the successful cultivators of their special department of learning. Thus it is, the future may be made to purge, and ultimately care for its own interests, while those now engaged in the medical profession in absence of other, will find their reward for unremitting labor in its culture, and in the consciousness of duty performed for the general weal. W. T.

